# Development of A Spectrometric Enzyme-Coupled Assay for 11-Aminoundecanoic Acid Transaminase and Its Detection in *Pseudomonas* Strain JG-B Cell Lysate

**DOI:** 10.17912/micropub.biology.002133

**Published:** 2026-06-03

**Authors:** Sophia Yager-Motl, Lana Segizbayeva, Hisako Masuda

**Affiliations:** 1 IU Kokomo, Kokomo, IN, US

## Abstract

11-aminoundecanoic acid is a photochemical degradation product of the synthetic plastic nylon 11. With increasing plastic waste in the environment, understanding the fates of nylon 11 degradation product is important. Previously, a bacterial strain with an ability to metabolically degrade 11-aminoundecanoic acid was isolated, but the enzymes that are involved in the metabolism of 11-aminoundecanoic acid has yet to be determined. In this work, we developed an enzyme-coupled assay to study transaminase activity with 11-aminoundecanoic acid and pyruvate as co-substrates. Using this method, transaminase activity was successfully detected in the cell lysate of *Pseudomonas* strain JG-B, grown on 11-aminoundecanoic acid.

**Figure 1. Coupled Reaction to Investigate the Catalytic Action of Putative 11-AUA Aminotransferase:** (A) Top: production of alanine from 11-aminoundecanoic acid and pyruvate, catalyzed by aminotransferase(s). Below: reaction between alanine and NAD ^+^ to form pyruvate and NADH by the catalytic action of alanine dehydrogenase. The production of NADH was measured by spectrophotometer by monitoring the change in absorbance at 340 nm. (B) Kinetic study of NADH formation by alanine dehydrogenase. Absorbance at 340 nm was measured by a spectrophotometer (C) Kinetic study of NADH formation by a coupled assay involving 11-AUA and cell lysate. 11-AUA, pyruvate and cell lysate (or boiled lysate) were incubated at room temperature for 20 minutes before addition of ADH and NAD ^+^ .

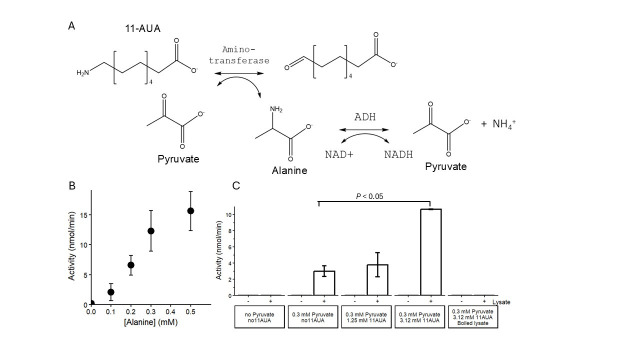



## Description

Nylon 11 is a synthetic polymer with high heat resistance, abrasion resistance, and elasticity (https://hpp.arkema.com/en/product-families/rilsan-polyamide-11-resins). Because of its desired physical properties and chemical resistance, it is widely used in a variety of commercial products such as oil prospection in the ocean, utility fibers, and medical devices (de Albuquerque Dias et al., 2024). 11 aminoundecanoic acid (11-AUA) has been shown as a chemical degradation product of nylon 11 polymers (Češarek et al., 2020). It is also speculated that 11-AUA is an intermediate of nylon 11 biodegradation by bacterial strains, as the bacteria isolated from the nylon 11 enrichment culture was capable of growing on 11-AUA as a sole source of carbon and nitrogen (Gatz-Schrupp et al., 2020).

For living organisms, the incorporation of nitrogen is crucial for synthesis of essential macromolecules such as nucleic acids, lipids and proteins. While common sources of nitrogen are amino acids for animals, microorganisms are capable of utilizing a variety of inorganic (e.g. NH _4_^+^ , NO _3_^-^ , and NO _2_^-^ ) (Arp and Stein, 2003), as well as organic nitrogenous compounds (Cook and Huetter, 1981; Hongwei et al., 2006; Weidhaas et al., 2009). For example, *Pseudomonas* strains utilize s-triazines as a nitrogen source (Cook and Huetter, 1981). *Rhodocossu opacus* strain can use 2,4,6-trinitrophenol, 2,4-dinitrophenol, and other nitroaromatic compounds as a sole nitrogen source (Weidhaas et al., 2009).

Transaminases (TAs), or aminotransferases, are important biocatalysts as they can synthesize chiral amines (Savile et al., 2010). Aminotransferases catalyze a transamination reaction that transfers an amino group to a ketoacid, ketone or aldehyde (Schiroli and Peracchi, 2015). The predominant amino group acceptors are α-ketoglutarate, oxaloacetate and pyruvate, producing glutamate, aspartate, and alanine, respectively (Schiroli and Peracchi, 2015).

Some ω-amino acids are building blocks of nylons and identified as an intermediate in known nylon oligomer biodegradation pathways (Takehara et al., 2018). For example, the metabolism of nylon 6 degradation involves a formation of ω-amino acids (6-aminohexanoate) as an intermediate (Yasuhira et al., 2007). Amino transferases that transfer amino group from these ω-amino acids have been identified (Kaulmann et al., 2007; Schrewe et al., 2013; Wilding et al., 2015). 6-aminohexanoate aminotransferase (NylD) of *Arthrobacter* sp. KI72 transfer amino group of 6-aminohexanoate to 2-oxoglutarate or glyoxylate (Takehara et al., 2018). Aminotransferase that transfers amino group from 12-aminododecanoic acid, which is a building block of nylon 12, to pyruvate were found in a *Pseudomonas* strain AAC (Wilding et al., 2015).

Previously, *Pseudomonas* sp. strain JG-B was isolated from a nylon 11 enrichment culture (Gatz-Schrupp et al., 2020). It exhibits a rapid growth in defined media that does not contain nitrogenous molecules other than an ω amino acid, 11-AUA. To the best of our knowledge, a step-by-step degradation pathway of 11-AUA has not yet been determined. In this study, to identify the fate of the amine group of 11-AUA in *Pseudomonas* sp. JG-B, we developed a spectrophotometric assay for aminotransferase that can utilize 11-AUA as a substrate.

We hypothesized that 11-AUA aminotransferase activity can be determined by coupling it with a reaction with alanine dehydrogenase (ADH), as described in Figure 1A . The transfer of amine group of 11-AUA to pyruvate is expected to be catalyzed by a putative 11-AUA aminotransferase(s), producing alanine as one of the products. The alanine will then react with NAD ^+^ and become converted to pyruvate and NADH by the action of alanine dehydrogenase.

First, using alanine standards, we confirmed whether the initial velocity of the ADH-catalyzed reaction exhibited a positive trend with the increasing alanine concentration ( Figure 1B ). NADH formation, as indicated by an increase in absorbance at 340 nm, was successfully detected when 0.1 U of alanine dehydrogenase and 1.25 mM NAD ^+^ were mixed with varying amounts of alanine.

Using this condition, ADH activity in a coupled reaction was tested. Initially, coupled reactions were allowed to undergo simultaneously by concurrently adding 11-AUA, pyruvate, NAD ^+^ , cell lysate and ADH to a reaction mixture. No detectable NADH production was detected. We then separated a coupled reaction into two separate steps (1) aminotransferase-catalyzed step, followed by (2) the addition of ADH and NAD ^+^ to initiate a time course measurement of absorbance at 340 nm. An increase in absorbance at 340 nm was observed when the reaction mixture of the first step was incubated for 20, 40 or 60 minutes. This suggests that accumulation of alanine produced by the first step is essential in allowing the second step to occur at the detectable level. Since there was no additional increase in absorbance with a longer incubation period, a 20-minute incubation period was selected and used for subsequent experiments.

Using this condition, production of NADH with and without 11-AUA, pyruvate and cell lysates were tested ( Figure 1C ). First, to eliminate the possibility that unknown compound(s) in cell lysates were the cause of absorbance increase, the following control experiment was performed. A reaction mixture was carried out without 11-AUA. Incubation of pyruvate and cell lysate devoid of 11-AUA yielded a small absorbance increase. This is most likely due to a reaction(s) between pyruvate and a nitrogenous molecule(s) in cell lysate, leading to alanine production. However, the NADH production was significantly higher when 3.25 mM 11-AUA was present. There was a statistically significant difference in the observed activity between a reaction with no 11-AUA vs. reaction with 3.25 mM 11-AUA ( *p* <0.05), strongly suggesting that the NADH production was due to the formation of alanine from 11-AUA and pyruvate by the action of an aminotransferase(s) in cell lysate. Second, to ensure that the observed increase in NADH was from an enzymatic reaction, this experiment was repeated with boiled lysate ( Figure 1C ). This did not yield a change in absorbance. Testing the 11-AUA concentration higher than 3.25 mM was impossible due to low 11-AUA solubility. When reaction contained smaller amounts (1.25 mM) of 11-AUA, NADH production was not at a level significantly higher than that of control ( Figure 1C ).

In this paper, we have described an enzyme-coupled reaction to analyze 11-aminoundecanoic acid aminotransferase. By coupling with alanine dehydrogenase catalyzed reaction, the production of alanine by the action of aminotransferase was detected spectrophotometrically. The assay was compatible with the use of cell lysate as the source of aminotransferase activity, as molecules and enzymes present in the lysate did not interfere with the reaction. The results of the experiments devoid of 11-AUA or the use of boiled lysate suggest that lysate did not yield an alternate substrate of alanine dehydrogenase and that an enzyme(s) in lysate is essential for NADH production by ADH. Our results also suggest that 11-AUA’s metabolic pathway most likely involves an aminotransfer reaction, yielding alanine and aldehyde product. The nature of this aminotransferase remains unknown. Further study will be conducted to identify and characterize enzymes involved in this and subsequent steps in *Pseudomonas* sp. strain JG-B.

In conclusion, we found that the cell lysate of strain JG-B, grown on 11-AUA as a sole source of carbon and nitrogen, produces aminotransferase(s) that yield alanine from 11-AUA and pyruvate. Our assay allows for an analysis of 11-AUA specific aminotransferase activity only requiring commonly available equipment.

## Methods

Spectrophotometric analysis was performed using UV-VIS spectrophotometer (Jenway 6715, Cole-Parmer, IL, USA). *Pseudomonas* sp. strain JG-B was grown on the modified basalts medium (BSM) (Hareland et al., 1975) devoid of carbon and nitrogen. Medium was supplemented by 0.4 mg/mL of 11-AUA as the sole source of carbon and nitrogen (Gatz-Schrupp et al., 2020). When the culture reached stationary phase, it was transferred to fresh media (100 mL) and grown until optical density at 600 nm reached 0.5. The cells were collected by centrifugation and were washed three times with lysis buffer consisting of 50 mM phosphate (pH 8.0) and 500 mM NaCl. The cells were lysed by freeze-thaw cycles after treatment with lysozyme at 37 °C for 30 minutes. Cellular debris and large insoluble materials were removed by centrifugation, and the supernatant (~ 3 mL) was designated as cell lysate.

Assay with alanine dehydrogenase using alanine and NAD ^+^ was performed as described before (Wilding et al., 2015). Between 0.1 and 0.5 mM of alanine was incubated with 12.5 mM NAD ^+^ with 0.1 U ADH in 50 mM phosphate buffer (pH 10). Absorbance at 340 nm was measured continuously for 200 seconds. The rate of NADH formation by alanine dehydrogenase was calculated using the extinction coefficient (6220 M ^-1^ cm ^-1^ ) of NADH. For a coupled reaction, designated amounts of 11-AUA were incubated with 0.3 mM pyruvate and 10 µL cell lysate for 20 minutes at room temperature before the addition of alanine dehydrogenase and NAD ^+^ . Experiments were performed in triplicates and *p* -value was calculated by the t-test.

## Reagents

All chemicals including 11-aminoundecanoic acid, pyruvate, NAD ^+^ , alanine, and alanine dehydrogenase (ADH) were purchased from Sigma-Aldrich (St. Louis, MO, USA). Deionized water was used to prepare solutions. *Pseudomonas* sp. strain JG-B was isolated previously in our laboratory.

## References

[R1] Arp Daniel J., Stein Lisa Y. (2003). Metabolism of Inorganic N Compounds by Ammonia-Oxidizing Bacteria. Critical Reviews in Biochemistry and Molecular Biology.

[R2] Češarek Urška, Pahovnik David, Žagar Ema (2020). Chemical Recycling of Aliphatic Polyamides by Microwave-Assisted Hydrolysis for Efficient Monomer Recovery. ACS Sustainable Chemistry & Engineering.

[R3] Cook Alasdair M., Huetter Ralf (1981). s-Triazines as nitrogen sources for bacteria. Journal of Agricultural and Food Chemistry.

[R4] de Albuquerque Dias Frederico Gonçalves, Veiga Amanda G., Gomes Antônio Pedro A. da C. P., Rocco Maria Luiza M., da Costa Marysilvia F. (2024). Recycling decommissioned polyamide 11: An approach to handle a previously unwanted material. Journal of Applied Polymer Science.

[R5] Gatz-Schrupp Jocelyn, Deckard Peter, Hufford Benjamin, Ly Steven, Tupa Peter, Masuda Hisako (2020). Isolation and genomic analysis of 11-aminoundecanoic acid-degrading bacterium
*Pseudomonas*
sp. JG-B from nylon 11 enrichment culture. Journal of Genomics.

[R6] Hareland W A, Crawford R L, Chapman P J, Dagley S (1975). Metabolic function and properties of 4-hydroxyphenylacetic acid 1-hydroxylase from Pseudomonas acidovorans. Journal of Bacteriology.

[R7] Hongwei Yang, Zhanpeng Jiang, Shaoqi Shi (2006). Biodegradability of nitrogenous compounds under anaerobic conditions and its estimation. Ecotoxicology and Environmental Safety.

[R8] Kaulmann Ursula, Smithies Kirsty, Smith Mark E.B., Hailes Helen C., Ward John M. (2007). Substrate spectrum of ω-transaminase from Chromobacterium violaceum DSM30191 and its potential for biocatalysis. Enzyme and Microbial Technology.

[R9] Savile Christopher K., Janey Jacob M., Mundorff Emily C., Moore Jeffrey C., Tam Sarena, Jarvis William R., Colbeck Jeffrey C., Krebber Anke, Fleitz Fred J., Brands Jos, Devine Paul N., Huisman Gjalt W., Hughes Gregory J. (2010). Biocatalytic Asymmetric Synthesis of Chiral Amines from Ketones Applied to Sitagliptin Manufacture. Science.

[R10] Schiroli Davide, Peracchi Alessio (2015). A subfamily of PLP-dependent enzymes specialized in handling terminal amines. Biochimica et Biophysica Acta (BBA) - Proteins and Proteomics.

[R11] Schrewe Manfred, Ladkau Nadine, Bühler Bruno, Schmid Andreas (2013). Direct Terminal Alkylamino‐Functionalization
*via*
Multistep Biocatalysis in One Recombinant Whole‐Cell Catalyst. Advanced Synthesis & Catalysis.

[R12] Takehara Ikki, Fujii Tsubasa, Tanimoto Yuuki, Kato Dai-Ichiro, Takeo Masahiro, Negoro Seiji (2017). Metabolic pathway of 6-aminohexanoate in the nylon oligomer-degrading bacterium Arthrobacter sp. KI72: identification of the enzymes responsible for the conversion of 6-aminohexanoate to adipate. Applied Microbiology and Biotechnology.

[R13] Weidhaas Jennifer L., Chang Daniel P. Y., Schroeder Edward D. (2009). Biodegradation of Nitroaromatics and RDX by Isolated
*Rhodococcus Opacus*. Journal of Environmental Engineering.

[R14] Wilding Matthew, Walsh Ellen F. A., Dorrian Susan J., Scott Colin (2015). Identification of novel transaminases from a 12‐aminododecanoic acid‐metabolizing
*P*
*seudomonas*
strain. Microbial Biotechnology.

[R15] Yasuhira Kengo, Tanaka Yasuhito, Shibata Hiroshi, Kawashima Yasuyuki, Ohara Akira, Kato Dai-ichiro, Takeo Masahiro, Negoro Seiji (2007). 6-Aminohexanoate Oligomer Hydrolases from the Alkalophilic Bacteria
*Agromyces*
sp. Strain KY5R and
*Kocuria*
sp. Strain KY2. Applied and Environmental Microbiology.

